# Implantable cardioverter defibrillators in paediatric patients: yet another example of healthcare divergence?

**DOI:** 10.1093/europace/euae230

**Published:** 2024-09-04

**Authors:** Elizabeth DeWitt, Jan Janousek, Susan P Etheridge

**Affiliations:** Department of Cardiology, Boston Children’s Hospital, Harvard Medical School, Boston, MA, USA; Children’s Heart Centre, Second Faculty of Medicine, Charles University in Prague and Motol University Hospital, Prague, Czech Republic; Boise St. Luke’s Pediatric Cardiology, Boise ID and Division of Pediatric Cardiology Department of Pediatrics, Stanford University, Stanford, CA, USA

As the world came together once again this summer in Paris for the Olympic Games, the viewing public had an opportunity to see the best athletes from around the globe compete on the world stage. The games historically have brought together athletes from all backgrounds to a common pitch, playing field, or pool deck. As with most things, however, the wealth of the nation represented and that country’s standards and expectations may influence the result on the field, in the pool, and at the track. The Olympic committee tried to minimize the environmental and climate impact of the games, one example in there being no air conditioning in units in the athlete’s village. However, wealthier countries like the USA and some European countries decided to purchase their own units from the organizers for their athletes’ rooms.^1^ Athletes from those countries had a cool room, no disadvantageous dehydration, or delayed recovery whereas athletes from smaller, poorer countries might have found themselves at a disadvantage even before the starting guns went off. Once again, the playing field was not equal.

The study from Thuraiaiyah *et al.* published in this issue of *Europace*, ‘Implantable cardioverter defibrillator therapy for paediatric patients for primary vs. secondary prevention,’^10^ provides another such opportunity for comparison of practices between countries, this time relating to the opportunity to examine management and approach to disease in one small country and compare it with broader trends in usage and approach in other areas of the world. The authors report a paediatric experience in implantable cardioverter defibrillator (ICD) utilization over a 40-year period from 1982 to 2021. They limited the inclusion to patients ≤15 years of age to target a ‘paediatric’ population. Implantable cardioverter defibrillators were implanted for both primary and secondary prevention, and for the typical indications and underlying pathology—cardiomyopathies, channelopathies with risk of arrhythmia, and in patients with congenital heart disease.

Denmark is a small country with population of around 5.9 million, in contrast to the US population of 333 million, or the entire European Union, encompassing around 448 million people. Over this 40-year period, 72 ICDs were implanted in the paediatric population described in this study, or an average of 1.8 implanted annually. The authors estimate their rate of implantation is 39 ICDs per million live births. In comparison, over a 6-year period that overlapped with this study from Denmark, in a study from the USA, 3461 ICDs were implanted in patients <21 years of age, or 577 ICDs annually.^2^ Using the reported birth statistics from 2016, this suggests that around 146 ICDs were implanted per million patients in patients in the USA, suggesting that the US rate of implantation is around ∼3.8 times that of Denmark. In contrast, an abstract presented at the Association for European Paediatric Cardiology in 2023 described the outcomes of paediatric patients (<18 years) with ICDs in the Czech Republic. The authors identified relatively similar rates of implant to that in Denmark—over a 30-year period from 1993 to 2022, 109 patients underwent ICD implant, a rate estimated to be around 34.6 ICD implants per million live births (abstract Cardiology in the Young 2023; vol 22 supplement S1 and personal communication). Beyond the USA and Europe, there are relatively few studies available for comparison; a study from Japan in 2014 described 64 ICDs implanted in patients <16 years of age between 1999 and 2012^3^ or an average of 5.3 annually; in 2010, a Japanese population census noted 1.07 million births that would suggest a rate of implant of 5 ICDs per million births. The literature is clearly weighted towards the experience in the USA and Europe, with even international multicentre studies drawing from these populations with occasional inclusion of Australia/New Zealand.^4^ There is minimal available literature regarding the use of ICDs in Africa, South America, or the Middle East.

In comparison to European countries, the USA implants twice as many ICDs for primary prevention in adult patients with hypertrophic cardiomyopathy^5^ and three times as many ICDs for primary prevention of adult patients with arrhythmogenic cardiomyopathy.^6^ It seems reasonable to infer that the USA may implant more devices for primary prevention in other populations with ICD indications as well. While there are no similar studies directly comparing rates of implant for these cardiomyopathies or inherited arrhythmia syndromes in a paediatric-specific population, it may also be reasonable to infer that the paediatric practice in each country might follow ‘local’ adult electrophysiology practices. Studies comparing the incidence of sudden unexpected death in paediatrics in both Denmark and the USA have found them to be similar—1.5 per 100 000 people in Denmark^7^ and 1.75 per 100 000 people in the USA^8,9^—thus, despite higher ICD use in the USA, there is no clearly decreased risk of sudden death.

What drives this more aggressive approach in the USA? It may be related at least in part to the structure of the US health system, driven by private health insurance as well as physician compensation structures that reward procedural medicine, in comparison with socialized medical systems where salaries may be comparatively fixed and independent from the relative value unit (RVU) type model. The culture of malpractice litigation in the USA may also lead to more devices placed for primary prevention. A country’s gross domestic product likely affects the uptake of such relatively expensive technology, but so also may impact the population’s attitude towards risk. In relatively stable societies where the risk of a young person dying from infectious disease, malnutrition, accidents, or war is very small, the incremental risk of death from a ‘preventable’ arrhythmia may be perceived to be more unacceptable than in places where threats of this nature are prominent. This too may drive higher rates of ICD implantation.

It is difficult to draw significant externalizable conclusions from this study based on the small study size, infrequent rate of implant, and the relatively homogenous population. It would be interesting to better understand the striking change in rate of implant for secondary prevention in the years 2015–19 and to understand what drove this change. This dramatic change reflected nearly twice as many implants of secondary prevention devices this 5-year period compared to the prior 15 years. Did the indications expand or the definition locally for secondary prevention change during this time, or was this truly just an anomaly? Paediatrics will always be limited by relatively small sample sizes relative to adult medicine, and generalizability of published single-centre experiences remains a challenge especially as seen in this article, there are clear divergent practices worldwide. While the authors in the present study strive to limit their analysis to a purely ‘paediatric’ cohort by limiting the upper age range to 15 years, there are limitations in this approach as similar studies have defined ‘paediatric’ differently. No doubt there would be more patients and more potential outcomes if one were to include patients 16–18 years or up to 21 years of age. Paediatrics may do well to attempt to adopt a more standardized approach to defining an at-risk population so that we may make more direct comparisons between smaller studies.

This article also speaks to the need for ongoing international collaboration between centres, not just between the USA and Europe but involving cardiologists and electrophysiologists around the world. Ongoing participation in organizations such as PACES (The Pediatric and Adult Congenital Electrophysiology Society) and their international arm, as well as local buy-in from institutions to support the data-sharing and institutional review board support are needed to develop meaningful international collaboration. Further collaboration and ongoing sharing of approaches to management for patients with these relatively rare conditions may ultimately improve the paediatric electrophysiology communities understanding of the phenotypes of these conditions and results of different approaches to management and containing risk. Ultimately, the goal is to have a ‘level playing field’ to provide excellent and data-driven care to patients regardless of where they live around the world.

**Conflict of interest:** none declared.
