# Correction to: Implantable cardioverter defibrillators in paediatric patients: yet another example of healthcare divergence?

**DOI:** 10.1093/europace/euae276

**Published:** 2024-11-06

This is a correction to: Elizabeth DeWitt, Jan Janousek, Susan P Etheridge, Implantable cardioverter defibrillators in paediatric patients: yet another example of healthcare divergence?, *EP Europace*, Volume 26, Issue 9, September 2024, euae230, https://doi.org/10.1093/europace/euae230

In the originally published version of this editorial the reference to paper being commented on was inadvertently omitted. The citation “ref” has been updated, and the reference list updated with a new reference 10.

